# Multifunctional Therapeutic Potential of Phytocomplexes and Natural Extracts for Antimicrobial Properties

**DOI:** 10.3390/antibiotics10091076

**Published:** 2021-09-06

**Authors:** Md. Mominur Rahman, Md. Saidur Rahaman, Md. Rezaul Islam, Md. Emon Hossain, Faria Mannan Mithi, Muniruddin Ahmed, Marianela Saldías, Esra Küpeli Akkol, Eduardo Sobarzo-Sánchez

**Affiliations:** 1 Department of Pharmacy, Faculty of Allied Health Sciences, Daffodil International University, Dhaka 1207, Bangladesh; mominur.ph@gmail.com (M.M.R.); mdsaidur569@gmail.com (M.S.R.); rezaul29-1301@diu.edu.bd (M.R.I.); emonhossain281033@gmail.com (M.E.H.); mithilafaria43@gmail.com (F.M.M.); drmuniruddin@gmail.com (M.A.); 2 Instituto de Investigación y Postgrado, Facultad de Ciencias de la Salud, Universidad Central de Chile, Santiago 8330507, Chile; marianela.saldias@ucentral.cl; 3 Department of Pharmacognosy, Faculty of Pharmacy, Gazi University, 06330 Ankara, Turkey; esrak@gazi.edu.tr; 4 Department of Organic Chemistry, Faculty of Pharmacy, University of Santiago de Compostela, 15782 Santiago de Compostela, Spain

**Keywords:** natural products, phytocomplexes, antimicrobial activity, alkaloids, tannins, terpenoids

## Abstract

Natural products have been known for their antimicrobial factors since time immemorial. Infectious diseases are a worldwide burden that have been deteriorating because of the improvement of species impervious to various anti-infection agents. Hence, the distinguishing proof of antimicrobial specialists with high-power dynamic against MDR microorganisms is central to conquer this issue. Successful treatment of infection involves the improvement of new drugs or some common source of novel medications. Numerous naturally occurring antimicrobial agents can be of plant origin, animal origin, microbial origin, etc. Many plant and animal products have antimicrobial activities due to various active principles, secondary metabolites, or phytochemicals like alkaloids, tannins, terpenoids, essential oils, flavonoids, lectins, phagocytic cells, and many other organic constituents. Phytocomplexes’ antimicrobial movement frequently results from a few particles acting in cooperative energy, and the clinical impacts might be because of the direct effects against microorganisms. The restorative plants that may furnish novel medication lead the antimicrobial movement. The purpose of this study is to investigate the antimicrobial properties of the phytocomplexes and natural extracts of the plants that are ordinarily being utilized as conventional medications and then recommended the chance of utilizing them in drugs for the treatment of multiple drug-resistant disease.

## 1. Introduction

Conventional medication has generally utilized an assortment of medicinal plants to treat drug-resistant diseases [1]. Old healers habitually joined helpful spices with odd spells, with plans and proprietary advantages passed down through the ages. Oral transmission of information about ancient restorative practices included modern conventions about plant gathering, readiness strategies, applications, portions, unique eating regimens, and linkages with other mystery tales about the idea of disease [2]. Plant species on the earth are assessed to number somewhere between 250,000 and 500,000 [3]. People and animals consume a limited quantity of them (1 to 10%). Quite possibly, significantly more are utilized for clinical purposes [4]. Hippocrates referenced 300 to 400 beneficial florae in the late 5th century Before Common Era (BCE) [5]. In the first century of the Common Era(CE), Dioscorides wrote De Materia Medica, a medicinal plant inventory that established the model for current pharmacopeias. Approximately 30 medicinal plants are described in the Bible. Indeed, the therapeutic virtues of frankincense and myrrh likely contributed to their high value. They were also used as mouthwashes because they were said to have antibacterial characteristics.

Western progression in the appreciation of helpful plants was hampered by the obliteration of urban foundations, with a critical record of plant drugs obliterated or lost. All through the Dark Ages, the Arab world proceeded to uncover and develop its previous signs. Asian societies, then again, were occupied with building their pharmacopeia. The Renaissance years in the West saw a resurgence of old medication, which depended for the most part on medicinal plants. Diseases have tormented people since the dawn of time, going from minor urinary parcel contaminations to massive pandemics. They utilized a broad scope of substances in their mending, including spices and other regular materials accessible around. For instance, a new report [6] found that nectar, a notable familiar substance, had antibacterial activity in vitro against *Staphylococcus aureus* and *Streptococcus pyogenes*. Numerous prescriptions have additionally been distinguished [7] because of ethnobotanical leads. The spice *Filipendula ulmaria* (sovereign of the knoll) was utilized to make the most significant pain-relieving and mitigating drug ibuprofen [8]. 

*Psoralea corylifolia*, *Catunaregum spinosa*, *Solanum virginianum*, *Woodfordia fructicosa*, and *Syzygium cumin* acetone extracts were proficient against the standard fungal culture of *Alternaria alternate* and *Fusarium oxysporum*. Inhibitory peptides for microbes were discovered in 1942 [9]. They have disulfide bonds and are frequently positively charged [10]. There is a scarcity of knowledge on antibacterial chemicals found in mushrooms [11]. Few chemicals that give antibacterial action to a variety of mushrooms have been discovered. The bulk of these chemicals are classified as terpenes. Phytotherapy is one of the oldest methods for the treatment of infectious disorders. Herbal medicine has a long history in many countries [12,13,14,15]. In flavonoid compounds, catechins, the most decreased sort of the C_3_ unit found, are especially significant. Due to their presence in oolong green teas, these flavonoids have been intensively studied. Teas have for quite some time been known to have antibacterial properties [9] and to contain a variety of catechin chemicals. *Vibrio cholerae* [16] *Streptococcus mutans* [17], *Shigella* [18], and other microbes and pathogens [19] were all suppressed in vitro by these compounds. Catechins hindered disconnected bacterial glucosyltransferases in S. freaks [20] and inactivated cholera poison in *Vibrio*, presumably because of the complexing exercises examined above for quinones. 

This exploration addresses the feasible execution of natural products in the treatment of infectious diseases. However, most of the previous studies have been performed on specific natural sources for screening potential antibacterial and/or antifungal properties. Considering the fact, this study was designed to assemble all the possible natural phytocomplexes, their chemical structures along with their potential bioactivity, and the mechanism of action against bacteria, fungi viruses, or protozoa, which could facilitate the approach of developing new antimicrobials from these inherent phytocomplexes [21].

## 2. Significant Gatherings of Antimicrobial Mixtures from Plants

**Figure 1:**
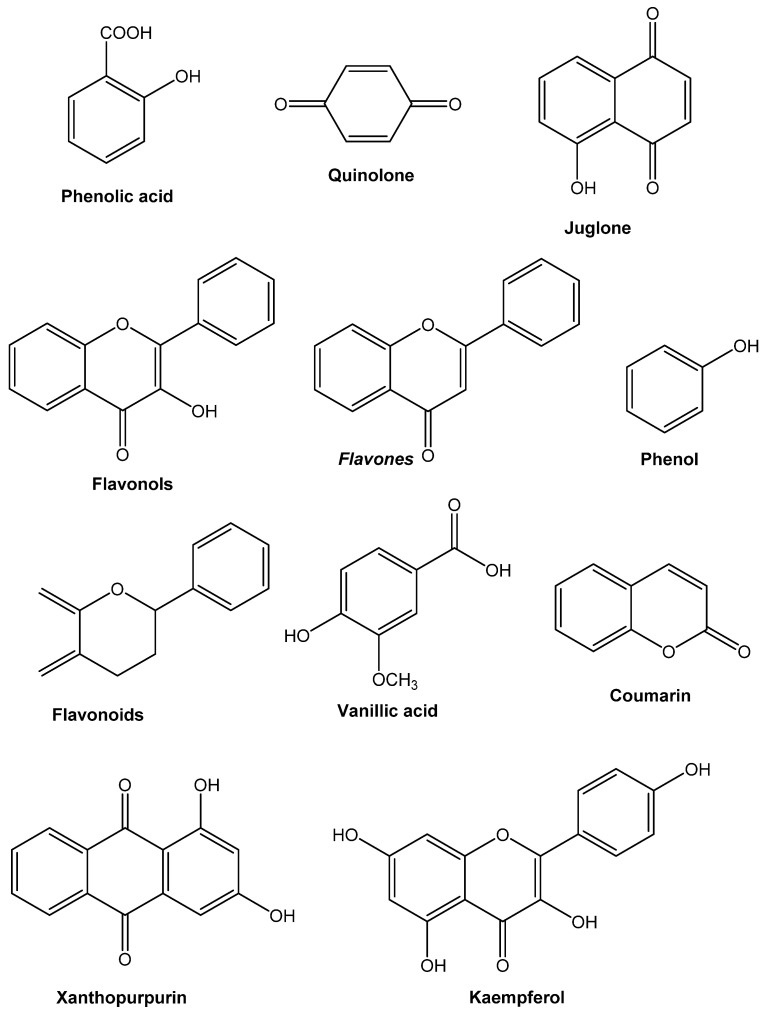
Secondary metabolites presented significant antimicrobial properties from medicinal plants and phytochemicals.

Plants can create a practically unlimited number of sweet-smelling compounds, most of which are phenols or their oxygen-subbed subsidiaries [22]. The more significant part is secondary metabolites (Figure 1), of which 12,000 have been recognized, representing under 10% of the total [23]. Plant smells are given by a few substances, for example, terpenes and terpenoids, although plant scent can be provided by others. Numerous atoms are liable for plant seasoning (for instance, the terpenoid capsaicin found in stew peppers). A few comparable plants and flavors used to prepare food by people likewise contain remedial substances [24].

### 2.1. Phenolics and Polyphenols

#### 2.1.1. Simple Phenols and Phenolic Acids

Verifiably the most un-problematic bioactive phytochemicals involve a single subbed phenolic ring. Caffeic and cinnamic acids are occurrences of various phenylpropane-construed manufactured subordinates with the best oxidation state. Caffeic acid, found in the common herbs tarragon and thyme, is efficient against viruses, bacteria [25,26], and fungi [27]. Pyrogallol and catechol are both hydroxylated phenols that have been demonstrated to be dangerous to minute organic entities. Pyrogallol contains three OH gatherings, while catechol has two. The measure of hydroxyl bunches on the phenol bunch and their location(s) on the phenyl bunch are thought to be associated with their overall harmfulness to microorganisms, with proof showing higher hydroxylation prompts expanded poisonousness [28].

Moreover, a few analysts have found that phenols that have been seriously oxidized are more inhibitory [29]. Compound restraint by oxidized synthetics, maybe by reactivity with sulfhydryl gatherings, or more vague communications with proteins are proposed to be liable for phenolic harmfulness to microorganisms. Essential oils with a C3 side chain are phenolic compounds which is less oxidized and have no oxygen, and they are now and again utilized as antimicrobials. Eugenol is present in clove oil, which is an outstanding delegate. The two growths [30] and microorganisms are considered bacteriostatic by eugenol. Phenol increases intracellular leakage, which includes the release of K+, which is the first sign of membrane breakdown [31] and radioactivity from 14C-labeled *Escherichia coli* [32]. According to Pulvertaft and Lumb [7], low dosages of phenols (0.032%, 320 g/mL) and other (nonphenolic) agents destroyed fast-developing cultures of *E. coli*, *staphylococci*, and *streptococci*, showing that autolytic enzymes were not included. According to Srivastava and Thompson [33,34], phenol only acts at the point where two daughter cells divide and that young bacterial cells are more vulnerable to phenol than older cells. Phenolics have antifungal and antiviral properties. The breakdown of the plasma membrane, which permits intracellular substances to spill out, is thought to be responsible for their antifungal action. Phenol has little effect on *Pseudomonas aeruginosa* PAO transduction by bacteriophage F116 has no impact on phage DNA within the capsid and has no impact on a variety of phage band proteins unless the treatment is 20 min or longer [35].

#### 2.1.2. Quinones

Quinones are shred rings that have two ketone replacements. They are discovered all through the world and are known for being incredibly receptive. Since these fabricated compounds are pigmented, they cause caramelizing in cut or injured foods from the ground and are a stage in the melanin arrangement passage in human skin [36]. They are available in henna, which gives it shading abilities [37]. The advancement from diphenol (or hydroquinone) to diketone (or quinone) through oxidation and diminishing reactions is essential. In various typical structures, the individual redox capacity of the quinone–hydroquinone pair is essential. Nutrient K is a naphthoquinone with a scramble construction. Its ability to oxidize in actual tissues may clarify its antihemorrhagic properties. Inside seeing fitting impetuses, for example, a polyphenol oxidase, hydroxylated amino acids can be changed over to quinones [37]. Quinones have been found to frame irreversible edifices with nucleophilic amino acids in proteins [38], leading to protein inactivation and function loss. As a result, quinone antibacterial effects have a broad range of applications. Surface-uncovered adhesins, cell divider polypeptides, and film-bound chemicals are generally expected targets in the microbial cell. Quinones may also make it difficult for bacteria to obtain substrates.

#### 2.1.3. Flavones, Flavonoids, and Flavonols

Flavones are widely distributed phenolic compounds with just a single carbonyl gathering (as opposed to quinones, which have two). A flavonol is created by adding a 3-hydroxyl group [37]. Flavonoids are hydroxylated phenolic compounds that are connected to an aromatic ring by a C_6_-C_3_ unit. It ought not to shock anyone that they have been discovered in vitro to be powerful antibacterial blends against a broad extent of microorganisms, given that they are notorious for being created by plants due to microbial contamination [39,40]. Their action is undoubtedly identified with their capacity to tie to extracellular and dissolvable proteins, just as to tie to bacterial cell dividers, as quinones do. Microbial films might be disturbed by more lipophilic flavonoids [41]. A catechin is a group of flavonoids that is present in a high concentration in green tea. Dental caries (created by *Streptococcus mutans*) were diminished by 40% when rodents were taken care of an eating regimen containing 0.1% tea catechins [42].

Flavonoid compounds inhibit multiple viruses. Flavonoids, including swertifrancheside [43], glycyrrhizin (from licorice) [44], and chrysin [45], are effective against HIV in numerous studies. Flavone derivatives have been shown to suppress respiratory syncytial virus (RSV) in several studies [46]. In vitro cell culture monolayers, Kaul et al. sum up the exercises and methods of quercetin, naringin, catechin, and hesperetin. While naringin was absent against herpes simplex ailment type 1 (HSV-1), poliovirus type 1, parainfluenza corruption type 3, and RSV, the other three flavonoids were successful. Hesperetin checked infectivity but not intracellular replication of the sum of the four pollutions; catechin hindered infectivity yet not intracellular replication of RSV and HSV-1; and quercetin bound infectivity yet not intracellular replication of the entire of the four ailments. Minimal hidden changes in the combinations, according to the makers, are vital for their ability, and many plant subordinates have an unimportant risky potential. The ordinary Western regular eating routine contains around 1 g of flavonoids and pharmacologically unique sums are presumably not going to hurt human hosts. The mechanism of action of flavones and flavonoids is challenging to pin down due to conflicting findings. Flavonoids with hydroxyl bunches on their rings are more dynamic against microorganisms than those without [47], suggesting that the membrane is their microbiological target. Lipophilic substances have a higher chance of disrupting this structure.

On the other hand, other authors have discovered that the higher the hydroxylation, the higher the antibacterial activity [48]. In a study with radioactive precursors, Mori and colleagues observed that flavonoids substantially restrict DNA synthesis in *Proteus vulgaris,* while RNA synthesis was most affected in *S. aureus*. The flavonoids that showed this activity were robinetin, myricetin, and (−)-epigallocatechin. Protein and lipid synthesis were also affected, but to a lesser extent. The B ring of flavonoids could be involved in intercalation or hydrogen bonding with the stacking of nucleic acid bases, according to the researchers, which could explain their inhibitory effect on DNA and RNA synthesis [49].

#### 2.1.4. Tannins

The explanation “tannin” infers a mix of polymeric phenolic builds that can tan calfskin or rush gelatin from the arrangement, a quality known as astringency. Their sub-nuclear loads an area from 500 to 3000, and they may be found in basically all pieces of the plant, including the bark, wood, leaves, ordinary things, and roots. Tannins are of two sorts: hydrolyzable and consolidated tannins. Hydrolyzable tannins are two or three esters of gallic damaging with glucose. Nonetheless, thick tannins (regardless, called proanthocyanidins) are incorporated flavonoid monomers. Tannins are comprised of flavan subordinates that have been moved to plant woody tissues and afterward consolidated. Tannins can likewise be delivered utilizing the polymerization of quinone units [50]. Since it was conjectured that drinking refreshments containing tannin, especially red wines and green teas, [51] would recuperate or forestall various afflictions, this gathering of synthetic substances has gotten a great deal of consideration as of late [52]. Tannins have been linked to multiple human physiological functions, including phagocytic cell activation, host-mediated tumor activity, and anti-infective capabilities [53]. Its synthetic capacities incorporate the creation of covalent securities with proteins by mysterious powers, for example, hydrogen holding and hydrophobic impacts, just as the development of covalent bonds [54]. As a result, their ability to inactivate microbial adhesins, enzymes, cell envelope transport proteins, and other proteins could be linked to their antimicrobial mode of action. They also form polysaccharide complexes [55]. The antibacterial potential of this activity has yet to be determined. Low tannin concentrations cause *Crinipellis perniciosa* germ tubes to change their form, indicating that bacteria are killed directly [56]. Tannins in plants impede insect development and ruminant animals’ digestive processes [57].

#### 2.1.5. Coumarins

Another phenolic intensifies, coumarins that contain intertwined benzene and pyrone rings [58]. At least 1300 had been identified as of 1996 [59]. Their antithrombotic, anti-inflammatory, and vasodilatory properties have made them famous. Warfarin is a prominent coumarin utilized as an oral anticoagulant comparatively as, oddly, a rodenticide. It could have antiviral effects. Coumarins are pretty dangerous in rodents. Hence, the clinical area around them is extremely cautious. The latest examination has uncovered an “articulated species-subordinate digestion,” which implies that numerous in vivo creature discoveries cannot be applied to people. In people, noxious coumarin subordinates give off an impression of being wiped out securely in urine [60]. A few more coumarins have antimicrobial properties. R.D. Thornes, a physician at Boston Lying-In Hospital, was looking for a way to cure vaginal candidiasis in his pregnant patients in 1954. *Candida albicans* was addressed to be curbed by coumarin in vitro. (The coumarin-spiked water supply was incidentally given to the entirety of the organisms in the assessment area achieving in vivo centers around rabbits. It also was shown to be an excellent defense mechanism when replicating endeavors began to come up short). Its estrogenic properties were found later [61]. The pharmacological effects of coumarins have been extensively described, and several of them have been discovered to have antibacterial activities. More recently, investigations have emphasized the potential importance of coumarins as alternative therapeutic methods, citing their capacity to reduce biofilm formation in clinically relevant bacteria and impede QS signaling systems (an intricate cell–cell communication system known as quorum sensing). In addition to human ailments, coumarins effectively manage plant pathogens, aquaculture infections, food spoilage, and lower biofouling created by eukaryotic organisms [62].

### 2.2. Terpenoids and Essential Oils

The Quinta essential, or fundamental oil division, conveys the aroma of plants. These oils are auxiliary metabolites with a high concentration of isoprene-based chemicals. Terpenes are diterpenes, triterpenes, and tetraterpenes (C_20_, C_30_, and C_40_), just as hemiterpenes (C_5_) and sesquiterpenes (C_5_), with an overall substance construction of C_10_H_16_ (C_15_). Terpenoids are synthetic substances that contain additional components, most normally oxygen. Terpenoids are comprised of acetic acid derivation units and consequently have a comparable beginning to unsaturated fats. They contrast with unsaturated fats in that they are cyclized and have a great deal of spreading. Methanol and camphor (monoterpenes) are instances of normal terpenoids, as are farnesol and artemisinin (polyterpenes) (sesquiterpenoids). Artemisin and its subsidiary α-arteether, also called by qinghaosu, discovered the present use as antimalarials [63]. In 1985, the controlling board of the logical working gathering of the World Health Organization chose to foster the last medication as a therapeutic for cerebral intestinal sickness. Bacteria [64], fungi viruses, and protozoa [65] are all susceptible to terpenes or terpenoids. In 1977, it was asserted that 60% of fundamental oil subordinates tried so far were growth inhibitors, while 30% were microorganism inhibitors [7]. Another triterpenoid betulinic corrosive was found to smother HIV. The exact mode of action of terpenes is unknown. However, it is thought that lipophilic molecules disrupt membranes. As a result, Mendoza et al. [66] discovered that adding a methyl group to the hydrophilicity of kaurene diterpenoids dramatically lowered their antibacterial action. Food experts have found that terpenoids found in plant essential oils can help control *Listeria monocytogenes* [67]. In the sterilization of lettuce leaves, oil of basil, a monetarily accessible natural, was discovered to be just about as powerful as 125 ppm chlorine [68]. The antibacterial activity of most terpenoids is linked to their functional groups, and the hydroxyl group of phenolic terpenoids and the presence of delocalized electrons are essential. Carvacrol methyl ether and p-cymene, for example, had much lower antibacterial activity than carvacrol. When the hydroxyl group in carvacrol is replaced with methyl ether, the hydrophobicity, antibacterial activity, and how it interacts with the membrane are all changed. Carvacrol’s antibacterial activity is equivalent to 2-amino-p-cymene, implying that the hydroxyl group is essential but unnecessary for carvacrol’s function. Various theories have been proposed to explain essential oils’ antibacterial properties. Essential oils destabilize cellular architecture and increase permeability, disrupting multiple cellular operations such as energy synthesis (membrane-coupled), membrane transport, and other metabolic regulatory functions. Many cellular processes, including energy synthesis (membrane-coupled), membrane transport, and other metabolic regulatory functions, are disrupted as a result. Energy conversion, nutrition processing, structural macromolecule synthesis, and growth regulator secretion may benefit from essential oils’ cell membrane disruption. Essential oils have the potential to alter both the cell’s external envelope [69] and its cytoplasm. Because essential oils are lipophilic, they can easily penetrate through bacterial cell membranes. Essential oils from various MAPs have increased bacterial cell membrane permeability, causing cellular component leakage and ion loss. Essential oils’ antibacterial activities are connected to reduced membrane potentials, disruption of proton pumps, and ATP depletion. This change in cell architecture could set off a chain reaction that affects other cell organelles. According to Cox et al., tea tree oil inhibits the growth of *S. aureus* and *E. coli* via altering cell permeability, increasing intracellular K+ ion leakage, and disturbing cell respiration. Essential oils disturb the arrangement of various fatty acids, phospholipid bilayers, and polysaccharides molecules as they move across the cell membrane and cytoplasmic membrane. The cytoplasmic coagulation of inner cellular components and the disintegration of the connections between the lipid and protein layers could be caused by any of these events [10].

### 2.3. Alkaloids

Alkaloids are basic nitrogenous compounds that are heterocyclic. In 1805, morphine was derived from the opium plant *Papaver somniferum*, the main therapeutically persuading alkaloid; the term morphine derives from the Greek word Morpheus, meaning leader of dreams. Both codeine and heroin are morphine aids. Antimicrobial effects have been found in diterpenoid alkaloids, customarily removed from plants in the Ranunculaceae, or buttercup family Solamargine, a glycoalkaloid present in the berries of *Solanum khasianum*, and various alkaloids have been exhibited to be faltering against HIV illness and AIDS-related intestinal contaminations. In contrast, alkaloids have been shown to have micro-biocidal properties (especially against Giardia and Entamoeba species, their significant antidiarrheal improvement is undoubtedly related to their impact on the little stomach-related bundle travel time. The alkaloid berberine is a critical individual from the alkaloid family. It can kill trypanosomes and plasmodia. The capacity of profoundly fragrant planar quaternary alkaloids like berberine and harmane to intercalate with DNA is believed to be the activity system. The antibacterial mode of action of alkaloids varies depending on the kind. The antibacterial activity of the indolizine alkaloids pergularinine and tylophorinidine are due to the inhibition of nucleic acid synthesis by the enzyme dihydrofolate reductase. Two mechanisms of bacterial inhibition have been discovered within the isoquinoline class: the Ungereminea phenanthridine isoquinoline inhibits nucleic acid synthesis, whereas studies with benzophenanthridine and protoberberine isoquinolines suggested that those agents act by disrupting the Z-ring and inhibiting cell division, which was investigated further. Agelasines alkaloids have an antibacterial action by inhibiting the dioxygenase enzyme BCG 3185c, which impairs bacterial hemostasis. This was discovered using overexpression and binding affinity experiments on the anti-mycobacterial alkaloid agelasine D. Saqualamine, a polyamine alkaloid, affects bacterial membrane integrity.

### 2.4. Lectins and Polypeptides

Inhibitory peptides for microbes were initially discovered in 1942. It is containing disulfide bonds and is frequently positively charged. The improvement of particle directs in the microbial film or cutthroat concealment of microbial protein bond to have polysaccharide receptors [70] could be their activity component. Against HIV, peptides and lectins have gotten a great deal of consideration as of late. However, these macromolecules, like those found in the herbaceous *Amaranthus*, have for some time been known to stifle microscopic organisms and parasites [71]. Thionins are peptides with corrosive amino deposits that are frequently identified in grain and wheat. Yeasts, just as Gram-negative and Gram-positive microscopic organisms, are noxious to them. Sugar beet thionins AX1 and AX2 are dynamic against improvement in any case not small life structures. Fabatin, a 47-advancement peptide from fava beans that suppresses *P. aeruginosa*, *E. coli*, *Enterococcus hirae* yet not *Saccharomyces* or *Candida*, shows up to be on a very basic level related with thionins from grains that limit *P. aeruginosa*, *E. coli*, *and Enterococcus hirae* at any rate, not *Candida* or *Saccharomyces.* Larger lectin molecules, such as mannose-specific lectins from various plants MAP30 from bitter melon GAP31 from *Gelonium multiflorum* and jacalin inhibit viral development (HIV, CMV) by inhibiting [9] viral interaction with key host cell components. Most general plant antimicrobial screening approaches, such as bioassay-guided fractionation procedures used by natural-products chemists, will overlook molecules and compounds like these whose mode of action could be to inhibit adhesion [16].

## 3. Some Phytochemicals That Have Antimicrobial Activity 

### 3.1. Kaempferol 

The juice of *Moringa oleifera* Lam*. Adenanthera pavonina* L. and *Annona squamosa* L. contain flavonoids, kaempferol, and quercetin, which possess potent antimicrobial properties [72]. Kaempferol is a small molecule with an anti-biofilm activity found to suppress the formation of *S. aureus* biofilms. Kaempferol inhibits bacteria’s adhesion to fibrinogen, which is the initial step in forming *S. aureus* biofilms. Kaempferol decreases surface protein anchoring through reducing SrtA function, according to one theory. Another theory is that kaempferol suppresses the expression of specific surface proteins. As kaempferol affects the attachment phase of biofilm development specifically, we identified genes connected to adhesion protein expression. Clumping factor, A and clumping factor B are the essential proteins for S. aureus to bind to fibrinogen, and they are encoded by the genes clfA and clfB. Fibrinogen-binding proteins ClfA and ClfB are up-regulated during biofilm formation in *S. aureus.* In addition to fibrinogen-binding proteins, *S. aureus* has two fibronectin-binding proteins, FnBPA and FnBPB, encoded by fnbA and fnbB, respectively. As they induce biofilm formation through a self-association process separate from ligand binding, FnBPs are multifunctional in the *S. aureus* biofilm life cycle [73].

### 3.2. Juglone 

For a long time, society’s medication has utilized the juice of newly macerated unripe bodies of the dark pecan (*Juglans nigra*) to remedy confined, parasitic skin infections like ringworm. It has been proposed that the organic movement of pecan bodies is identified with the presence of juglone (5-hydroxy-1,4 naphthoquinone), a basic naphthoquinone disconnected by sublimation from the unripe hulls. *S. aureus* is restrained by this anti-microbial compound, which ties to DNA and disturbs cell divider arrangement, putting bacterial cells under higher peroxidative pressure [74]. Juglone is antimicrobial, and it is thought to inhibit Gram-positive bacteria like *S. aureus*. Juglone has a particular antibacterial effect against distinct types of *S. aureus*. In the same bacterium, juglone has been postulated as a natural source of antibiotic resistance-modifying activity and a phytochemical element with antibiotic resistance-modifying activity. It is also probable that redox cycling is involved in juglone’s antifungal and antibacterial properties. According to bioinformatics analysis, juglone inhibits bacterial proteins involved in DNA, RNA, and protein production, as well as the tricarboxylic acid cycle. Juglone nanoparticles have also been demonstrated to have better antifungal and antibacterial activity than free juglone, showing that nanoparticle production could be a viable technique for enhancing juglone’s beneficial effects [75].

### 3.3. Xanthopurpurin and Vanillic Acid 

In children under the age of five, rotavirus infections are the most common cause of dehydrating gastroenteritis. Despite universal rotavirus immunizations, it remains a leading cause of death, particularly in poverty-stricken countries (94). These infections contaminate enterocytes and cause diarrhea by reducing enterocyte absorption capacity, expanding intestinal emission set off by viral non-underlying protein 4, and initiating the enteric sensory system [76]. 

In MA104 cells, *Rubia cordifolia* L. extracts and identified chemicals such as *vanillic acid* and *xanthopurpurin* were very efficient against rotavirus. By enhancing virus-mediated apoptosis, they were able to suppress its proliferation [77]—vanillic acid (VA) antibacterial activity and mechanisms of action against carbapenem-resistant *E. hormaechei* (CREH). VA’s antibacterial effectiveness against CREH was investigated using variations in intracellular ATP concentration, intracellular pH, membrane potential, and cell shape. The usefulness of VA in reducing biofilm formation and inflicting VA damage to CREH cells trapped in biofilms was also examined. A decline in intracellular ATP, pH, and membrane potential, as well as specific alterations in cell shape, suggest that VA may affect CREH’s cell membrane integrity. VA also demonstrated a considerable inhibitory effect on CREH biofilm formation and killed CREH cells within biofilms. VA disrupts the cell membrane of carbapenem-resistant *E. hormaechei.* Carbapenem-resistant VA inhibits the formation of *E. hormaechei* biofilms and kills carbapenem-resistant *E. hormaechei* cells within biofilms [78].

## 4. Antimicrobial Activity of Medicinal Plants

Plant-determined antimicrobials have amazing supportive assurance. They are capable of treating powerful sicknesses while also avoiding countless horrible effects that fabricated antimicrobials are known. Plant materials’ positive therapeutic effects are, for the most part, a direct result of a mix of assistant blends found in the plant. Auxiliary metabolites in plants and fundamental oils like alkaloids, tannins, steroids, phenol compounds, flavonoids, steroids, unsaturated fats, gums, and tars are suitable for conveying a specific physiological outcome to the living being (Table 1 and Table 2). Mixtures obtained from various segments of the plants can be utilized to treat looseness of the bowels, diarrhea, and different diseases. Blends from different segments of the plants can be used to deal with ailments like looseness of the bowels, diarrhea, hack, cold, cholera, and fever. *B. cereus*, *S. aureus*, *E. coli*, *C. albicans*, and *P. aeruginosa* were utilized to determine the antimicrobial activity of unrefined ethanolic concentrates of 10 medicinal plants used in conventional medication against five microorganisms *B. cereus*, *S. aureus*, *E. coli, C. albicans*, and *P. aeruginosa*. Five out of these ten plants analyzed and expressed antibacterial activity against at least one organism animal category. *Sanguisorba officinalis*, *Chelidonium majus*, and *Tussilago farfara* were potent antibacterial herbs. Nine plants were assessed for antibacterial properties. *Hibiscus rosasinensis*, *Sapindus emarginatus*, *Mirabilis jalapa*, *Nyctanthes arbortristis*, *Rheo discolor*, *Colocasia esculenta*, *Gracilaria corticata*, *Dictyota* sp., and *Pulicaria wightiana* were among the plants examined. *Pseudomonas testosteroni, Staphylococcus epidermidis, Klebsiella pneumoniae*, *Bacillus subtilis*, *Proteus morganii*, and *Micrococcus flavus* were evaluated for antibacterial activity. The activity of these plants was investigated following two methods: Agar disc diffusion and Agar plug diffusion. The bacterium strains *P. testosteroni* and *K. pneumoniae* were expressed as the most resistant. 

Several diseases, including asthma, gastrointestinal complaints, skin disorders, respiratory and urinary troubles, and hepatic and cardiovascular illness, have long been treated with medicinal herbs [79,80]. These plants create various physiologically active compounds [81,82] that are required for them to survive and prosper in the environment, including defensive responses to abiotic challenges such as temperature, water availability, mineral nutrition, and insect pests [81,82,83]. Physiologically active compounds in medicinal plants vary widely depending on plant species, soil type, and microbial interaction [84,85]. The bioactive secondary metabolites of medicinal plants have been shown to have a considerable impact on plant-associated microbial populations and physiological activities [86,87,88,89]. Growth promotion, nutrient acquisition, induced systemic resistance, and abiotic stress tolerance are all traits and actions for which plants rely on their microbiome [89,90,91,92,93,94]. Despite substantial research into the phytochemical contents and pharmacological activity of a wide range of therapeutic plants, the microbiome and physiological interactions between the host and microbes remain poorly understood. Several microbial communities dwell in the roots, shoots, and endospheric of plants, forming the microbiome [94,95]. The rhizospheres of many plants have been studied and found to be a good source for choosing beneficial bacteria that can improve plant health [87,96,97]. Understanding how microbial communities adapt to changes in the physiochemical environment of the rhizosphere could help researchers better understand the microbial ecology of plant-associated microorganisms. [89] Researchers observed a significant density of antagonistic bacteria in the rhizosphere of the therapeutic plants *Matricaria chamomilla*, *Calendula officinalis*, and *Solanum distichum*. The root-associated bacteria of *Ajuga bracteosa* boosted plant growth in a variety of ways by producing siderophores and indole acetic acid, as well as displaying antioxidant activity [98]. Plant phytochemical constituents are thought to be linked to endophytic bacteria and their interactions with host plants, either directly or indirectly [86,99,100]; it is thought that plant phytochemical constituents are linked to endophytic bacteria and their interactions with host plants, either directly or indirectly [101].

**Table 1:** Plants with antimicrobial characteristics have been utilized in traditional medicine mL [102].

Scientific Name	Plant Parts	Active Principle	Antimicrobial Activity	MIC Value
*Aloe vera*	S	Latex	*Salmonella* and *Streptococcus* spp.	*P. aeruginosa:* ≤400 μg/mL [103]
*Malus sylvestris*	FR	‘Phloretin’ a flavonoid	General antimicrobial	*P. aeruginosa:* 7.81 μg/mL [104]
*Withania somnifera*	R and L	‘Withaferin A’ a lactone	Antibacterial Antifungal	*S. aureus*: 250 μg/mL [105]
*Aegel marmelos*	L, FR and R	Essential oils/terpenoids	Antifungal	*A. fumigatus:* 15.625 μg/mL
*Barberis vulgaris*	R and SB	‘Barberine’ an alkaloid	*M. tuberculosis,* *Vibrio cholera,* *Plasmodium* and *Trypanosomes*	*P. aeruginosa:* 16 μg/mL *Proteus vulgaris:* 32 μg/mL *E. coli:* 32 μg/mL [106]
*Ocimum sanctum*	a. L and Sb. R	a. Essential oils b. Root extract	a. *Salmonella, Ringworm,* common cold virus b. In malarial fever to bring sweating	*S. aureus:* 128 μg/mL [107]
*Laurus nobilis*	Leaves	Essential oils	Antibacterial and antifungal	*E. coli:* >22.5 mg/mL, *P. aeruginosa:* 22 mg/mL [108]
*Piper nigrum*	S	‘Piperine’ an alkaloid	Fungi, Micrococci, *E. coli*	*C. albicans:* 3.125 mg/mL [109]
*Bacopa monnieri*	WP	‘Brahmine’ an alkaloid	Anthelmintic property	*UTI* and *RTI* bacteria: 2.5 mg/mL *E. coli:* 2.5 μg/mL [110]
*Acorus calamus*	RH and L	Volatile oils	Enteric bacteria Insecticidal	*S. aureus* and *E. coli*: 5–10 mg/mL
*Ricinus communis*	S	Castor oil	Antifungal (in dermatitis)	*S. aureus:* 62.5 μg/mL [111]
*Cinnamomum verum*	BA and L	Essential oils	General antimicrobial	*S. aureus:* 0.5 μL/disc [112]
*Cinchona officinalis*	BA	‘Quinine’ an alkaloid	Antimalarial	*Helicobacter pylori:* 0.1 ng/ML [113]
*Capsicum annum*	FR	‘Capsaicin’ a terpenoid	Antibacterial	From 10 to 20 μg/mL [114]
*Hydnocarpus kurzii*		Essential oil	*Mycobacterium leprae*	Thyme: 1.25 mg/mL [115]
*Coriandrum sativum*	WP, L, S		Antibacterial Antifungal	*Candida albicans:* 0.02 mg/mL, *E. coli:* 0.64 mg/mL [116]
*Eucalyptus globulus*	L	Tannins and terpenoids /essential oils	Antibacterial Antiviral Antifungal	*S. aureus*: 64 mg/mL, *S. pyogenes:* 32 mg/mL, *S. pneumoniae*: 16 mg/mL, *Haemophilus influenzae:* 16 mg/mL [117]
*Allium sativum*	B	Sulfated terpenoids	General antimicrobial	(Methanolic extract) *S. aureus:* 1.25 mg/mL *S. pneumonia:* 0.312 mg/mL *P. aeruginosa:* 1.25 mg/mL *K. pneumoniae*: 0.312 mg/mL(Ethanolic extract) *S. aureus:* 2.5 mg/mL *S. pneumonia:* 0.312 mg/mL *P. aeruginosa*: 0.625 mg/mL *K. pneumoniae:* 0.156 mg/mL [118]
*Piper longum*	FR and L	Piperin	Antibacterial	*B. cereus* and *E. coli*: 12.5 mg/mL [102]
*Glycyrrhiza glabra*		1. ‘Glycyrrhizin’ a terpenoid 2. ‘Glabrol’ an alcohol	1. HIV virus and other viruses 2*. M. tuberculosis* *S. aureus*	*S. aureus*: 50 mg/mL [119]
*Calendula officinalis*	L FL	Essential oils/Terpenoids	General antimicrobial	*S. mutans*: 3.12 μg/mL [120]
*Mentha arvensis*	L	‘Menthol’ an alcohol	AntisepticMouth wash	*Acinetobacter baumannii*: 23.5 μg/mL [121]
*Azadirachtus indica*	R, LB, FR, FL	1.‘Azadirachtin’ 2. ‘Nimbin’ 3. ‘Nimbidin’ 4. ‘Gedunin’ 5. ‘Salannin’ 6. ‘Quercetin’	1. Repellant and antifeedant 2. Antifungal 3. Antibacterial, antifungal 4. Anti-malarial, antifungal 5. Repellant 6. Antibacterial, antiprotozoal	*Streptococcus* sp.: 125 μg/mL *S. aureus:* 250 μg/mL *Enterococcus faecalis:* 500 μg/mL [122]
*Oleo europoea*		‘Hexanal’ an aldehyde	General antimicrobial	*S. cerevisiae*: 24 μg/mL, [123]
*Allium cepa*	B	‘Allicin’ a sulfoxide and other sulfated terpenoids	Antibacterial Antifungal	*S. aureus:* 7 μg/mL [124]
*Citrus sinensis*	FP, L	Terpenoids	Antifungal	*Streptococcus* sp.:12.4 mg/mL
*Carica papaya*	LA, FR	Terpenoids, organic acids, and alkaloids	General antimicrobial	*S. aureus*: 1250 μg/mL
*Butea monosperma*	S, L	Tannins	Round worm, Ring worm, Dhobi-itch	*Acinetobacter* sp: 2.62 mg/mL [125]
*Mentha piperita*	WP	1. ‘Menthol’ an alcohol 2. Peppermint oil terpenoid	1. General antimicrobial 2. Mouth freshener	*Klebsiella pneumonia:* 0.4 ± 0.02(*v/v*) [126]
*Papaver somniferum*		‘Opium’ an alkaloid	General antimicrobial	*K. pneumonia:* 2.2 mg/mL *C. albicans*: 1.1 mg/mL [127]
*Solanum tuberosum*	T	Potato starch	Antibacterial Antifungal	*S. aureus*: 0.62 mg/mL, *S. pyogenes*:1.25 mg/mL [101]
*Rauwolfia serpentine*	R	‘Reserpine’ an alkaloid	General antimicrobial	*S. aureus*: 30 mg/mL [101]
*Pterocarpus santalinus*	W	Terpenoids	Antibacterial, Antiseptic Against skin infections and inflammations	*S. aureus*: 4 mg/mL
*Catharanthus roseus*		Ajmalicine, serpentine, reserpine (alkaloids)	General antimicrobial Anti-cancer	*E. coli:* 12.5 µg/mL
*Santalum album*	W	Terpenoids, saponins, phenolic compounds	Antibacterial Skin infections TB of gallbladder	*S. aureus*: 0.078 µg/mL [128]
*Centratherum anthelmintium*	S		Anthelmintic	*E. coli:* 0.0020 µg/mL *P. aeruginosa*: 0.006 µg/mL
*Sida cardifolia*	WP and R with ginger		Antimicrobial	*C. albicans:* 8.33 µg/mL
*Thymus vulgaris*		‘Caffeic acid’, ‘thymol’ and tannins	Antibacterial, antiviral, antifungal	*S. aureus*: 0.312 mg/mL
*Tamarindus indica*	PF		GIT infections and toxicity	*E. coli*: 15 mg/mL and *Shigella flexnerri*: 10 mg/mL [129]
*Curcuma longa*	R, RH and L	‘Curcumin’, turmeric oil, terpenoids	Antibacterial, antiprotozoal, Anthelmintic	*S. aureus:* 190 mg/mL
*Salix alba*		‘Salicin’, tannins, and essential oils	General antimicrobial	*S. aureus*: 100 mg/mL
*Gaultheria fragrantissima*		Tannins and polyphenols	Hook worms, mosquito and fly repellant, anticancer drug	Inhibited growth and aflatoxin B1: 7 at 1.0 and 0.7 µL/mL, respectively [130]
*Artemisia maritime*	Immature F and L		Anthelmintic (worms and round worms), GIT infections	*B. subtilis, S. aureus*, *Salmonella* sp: 0.09mg/mL [131]
*Terminalia chebula* *Terminalia belerica Embilica officinalis*	S and FR		*S. aureus* *E. coli* *P. aeroginosa* *M. tuberculosis* Common cold virus	*S. typhimirium*: 1 mg/mL, MRSA: 0.25 mg/mL [132]

AE: Aerial part; B: Bulb; BA: Bark; FL: Flower; FP: Fruit peels; FR: Fruit; L: Leave; LA: Latex; LB: leaves bark; R: Root; RH: Rhizome; S: Seed; SB: Stem bark; PF: Pulp of fruit; W: Wood; WP: Whole plant; T: Tuber.

**Table 2:** Effect of essential oil on the pathogenic microorganisms [133].

Plant	Part Used	Chemical Compounds	Inhibited Microorganisms
*Cymbopogon citrates* *Allium sativum*	FR B	Ethanolic compounds Isothiocyanate	Enterobacteriaceae, *S. aureus* Enteriobacteriacae, *Candida* spp. [134]
*Thymus vulgaris*	AE	Thymol, Linalol, Carvacrol	*L. monocytogens, E. coli,* *S. typhimirium, S. aureus* [134]
*Pimpinella anisum*	S	Trans-anethole	*S. typhimirium, E. coli* [135]
*Origanum vulgare*	AE	Carvacrol, Thymol, γ-Terpinene	*L. monocytogens, E. coli,* Adeno virus, Polio virus [136]
*Feoniculum vulgare*	S	Trans-anethole	*Alternaria alternata, Fusarium oxysporium, Aspergillus flavus* [137]
*Cinnamomum zeylanicum*	BA	Cinnamaldehyde	Enterobacteriacae [138]
*Amomum kerervanh*	S	Ethanolic compounds	Enteriobacteriacae [139]
*Syzygium aromaticum* *Zingiber officinale*	FB RH	Eugenol, Eugenylacetate Ethanolic compounds	Enteriobactericae *A. fumigatus, Candida* spp., Adeno virus, Polio virus [140]
*Artemisa arborescens*	L	β-Triketone	*Herpes simplex* virus [141]
*Rosmarinus officinalis*	FL	Benzaylacetate, Linalool, α-pipene	*E. coli, S. typhimirium,* *B. cerus, S. aureus* [142]
*Thymus vulgaris,* *Mentha piperita*	AE	1,8- Cineole, Eugenol	*S. aureus, S. typhimirium* *Vibrio parahaemolyticus* [143]
*Salvia officinalis*	AE	1,8-Cineole, α-pipene	*S. aureus, E. coli* [144]
*Verbana officinalis*	AE	Borneol, Geranoil	*S. aureus, E. coli, S. typhimirium,* *L. monocytogens* [145]

AE: Aerial part; B: Bulb; BA: Bark; FB: Flower bud; FL: Flower; L: Leave; RH: Rhizome; S: Seed.

## 5. Antifungal Activity of Medicinal Plants

*Catunaregum spinosa*, *Woodfordia fructicosa*, *Psoralea corylifolia*, *Solanum virginianum*, and *Syzygium cumin* (CH_3_)_2_CO extricates were discovered to be efficacious against the standard infectious culture of *Alternaria substitute* and *Fusarium oxysporum*. The growth of Fusarium oxysporum was inhibited by all of the plant extracts tested. *Psoralea corylifolia* has the most current inhibition, followed by *Calunaregum spinosa*. Except for *Syzygium cumini*, all plant extracts were shown to suppress Alternaria growth. *Catunaregum spinosa* and *Solanum virginianum* extracts showed the most inhibition [146]. Himalayans utilized lipophilic (Dichloromethane) leaf extracts of medicinal plants, which were studied. In bioautography, *Alternaria alternata* and *Curvularia lunata* were utilized as test living things. It was discovered that 5 of the 12 plant species expressed antifungal activity. To deliver silica gel TLC plates, CH_3_OH (1:9, *v/v*) was utilized as a dissolvable. Lipophilic concentrates of *Vitex negundo* (RF esteem 0.85), *Ipomea carnea* (RF esteem 0.86), *Thuja orientalis* (RF esteem 0.80), and *Cinnamomum camphora* (RF esteem 0.80) developed clear inhibitory zones (RF esteem 0.89). *Thuja orientalis* lipophilic leaf extricate has the best antifungal action. The agar strong dispersion strategy was utilized to describe distinctive natural and watery concentrates of leaves of *Indigofera suffruticose* (Fabaceae) for 17 contagious strains. Except for the liquid concentrate of leaves of *Indigofera suffruticosa* delivered by imbuement, most of the concentrates were sans antifungal. *Dermatophyle* strains had MIC upsides of 2500 mg/mL against Trichophyton rubrum and *Microsporum canis*.

## 6. Antimicrobial Fractions or Compounds Isolated from *Agaricus bisporus*

There is a scarcity of knowledge on antibacterial chemicals found in mushrooms [11]. Few chemicals that give antibacterial action to a variety of mushrooms have been discovered. The bulk of these chemicals are classified as terpenes. However, just a little research has been done to look into the chemicals responsible for *A. bisporus’* antibacterial activity. In recent years, intriguing results on potential elements that play a role in antibacterial activity in *A. bisporus* have been added to the current knowledge. Antibacterial efficacy of aqueous total protein extracts of grown *A. bisporus* was demonstrated, notably against methicillin-resistant *S. aureus* (MRSA) and *S. aureus* [147]. The entire protein was fractionated into three fractions, one of which has antibacterial properties. A DEAE-A50 ion-exchange column with a stepwise salt slop elution was used to purify this fraction further. SDS-PAGE revealed that the fraction produced an almost pure protein fraction. This protein’s molecular weight was calculated to be 22,500 Dalton. The MIC_50_ of this pure peptide against *S.aureus* and MRSA was 100 g/mL [147] 2,4-dihydroxybenzoic, and protocatechuic acids, which have previously been identified from numerous wild mushroom species, including *A. bisporus*, exhibited significant antibacterial activity in the search among phenolic compounds. 2,4-dihydroxybenzoic and protocatechuic acids were found to have antibacterial activity (MIC = 1 mg/mL) against Gram-negative bacteria such as *E. coli*, *Pasteurella multocida*, and *Neisseria gonorrhoeae* in clinical isolates [148]. However, no clear link between these phenolic chemicals and *A. bisporus* has been shown. It has been recently reported that fractionation of the grown *A. bisporus* methanol-dichloromethane (1:1) extract. Over 200 initial eluates were eluted using step-wise gradient elution, with six distinct fractions produced depending on their thin layer chromatography band patterns. Antibacterial activity was measurable and dose-dependent in one of the fractions (eluted by two eluent systems: ethyl acetate and ethyl acetate/methanol 1:1). While the crude extract of *A. bisporus* exclusively inhibited Gram-positive bacteria, the fraction inhibited both Gram-positive and Gram-negative bacteria, especially *E. coli,* with an MIC_50_ of 8 mg/mL. These discoveries might be a first step in determining the chemical structures of components that give *A. bisporus* its antibacterial properties [149,150]. Antifungal activity has been found in 52 species, the majority of which are edible mushrooms (*A. bisporus*), as far as we know. Even though researchers are less interested in non-edible mushrooms than edible mushrooms, they have different metabolites used in pharmaceutical products. As the majority of antifungal mushrooms are wild, there is more variance among the species evaluated. Mushroom cultivation demands a lot of unusual and difficult-to-find circumstances. Several factors influence mushroom cultivation, including meteorological and physiological circumstances and the prevalence of epidemics. Soil conditions affect the phenotype of all mushrooms, according to Pinna et al. [151], but each species has a different reaction. Soil moisture can either stimulate (*Boletus edulis* and *Lactarius deterrimus*) or delay (*Cortinarius caperatus and Catathelasma ventricosum*) the initial fructification stage. Insects, mites, crustaceans, and other arthropods that breakdown synthetic substrates or wood used in mushroom cultivation have also been found to stymie mushroom growth. Another study discovered that adding sodium carbonate precipitate (CaCO_3_) to the substrate enhances shiitake mushroom (*Lentinus edodes*) yield and size. Only a few species are created as a result of the aforementioned concerns, and the only way to obtain a varied range of mushrooms is to pick them in their natural habitats. Only twenty-one species are saprotrophic, which means they digest dead organic matter and are essential to the ecosystem’s balance. Sixteen species are mycorrhizal parasites, which infect a host and profit from a symbiotic relationship with the roots of plants and trees. Six are biotrophic parasites, which infect a host and benefit from a symbiotic relationship with the roots of plants and trees. The remaining five species are saprotrophic but also mycorrhizal [152]. Four species are necrotrophic parasite fungi that kill the host and then feed on the dead materials, finally becoming saprotrophic. Biologically active natural compounds can be found in plants, mushrooms, and other natural sources. Mushrooms require antibacterial and antifungal compounds to survive in their natural environments. As a result, antifungal compounds of varied potencies might be isolated from a range of mushroom species, potentially beneficial to humans [153]. Pathogenic fungus damage humans, agricultural animals, crops, and other species. Fungal infections can be life-threatening, wreaking havoc on one’s health and finances. The organism has an intrinsic ability to fight fungal invasions by producing antifungal compounds; however, this ability is weakened in immunocompromised individuals, and fungal infections become more common. In addition, fungal invasion in agriculture causes considerable crop quality and productivity reductions, as well as large economic losses. Antifungal chemical research could lead to remedies, such as introducing genes expressing antifungal proteins into crops to boost their resistance to fungal infections [154,155].

## 7. Phytotherapy

Phytotherapy is one of the oldest methods for the treatment of infectious disorders. Herbal medicine has a long history in many countries [14,156,157]. Phytotherapy makes use of whole plants or portions of plants that have been prepared in various ways. Oils, colors, plant eliminates, mother tone (TM), essential oils, suppositories, syrups, inhalants, and different mixes are among the many plant things available today. There have been considered that not just show that specific plants have antibacterial properties but also pinpoint the possible objective of the activity. *Juglans regia* (walnut) related to *Camellia sinensis* (tea bush) hinders numerous opposition microbes (MDR) by acting synergistically with diverse antimicrobial mixtures, most likely assaulting the cell wall of bacteria [158].

### 7.1. Phytotherapy in Bacterial Infection 

#### 7.1.1. Respiratory Tract Infection 

*Cinnamomum zeylanicum* (cinnamon), *Daucus carota* (wild carrot), *Eucalyptus globulus* (eucalyptus), and *Rosmarinus officinalis* (rosemary) essential oil mixes are additionally valuable in treating influenza sickness and bacterial challenges [159]. Chemical substances such as carvacrol, cinnamaldehyde, eugenol, and camphor have been identified as the key chemical components responsible for antibacterial activity in their essential oils [160,161,162].

The antibacterial actions of Eos are thought to work by inflicting structural and functional damage to the bacterial cell membrane [163].

#### 7.1.2. Urinary Tract Infection

Most uropathogenic strains are inhibited or prevented by aqueous extracts of *Calluna vulgaris* (common heather) and *Vaccinium vitis-idaea* (lingonberry, cowberry, or bearberry [164]. The antibacterial effects of various *C. vulgaris* extracts revealed that phenolic compounds and flavonoids were responsible for bacterial strain growth inhibition.

The aqueous extract of *C. vulgaris* showed significant antibacterial activity against different strains of *E. coli, E. faecalis*, and *P. vulgaris* in an in vitro test. The MIC values for this extract ranged from 2.5 mg/mL to 20 mg/mL [165].

#### 7.1.3. Cutaneous Infection

The utilization of skin therapeutics with various plants or plant–drug blends is typical. A couple of in vivo and in vitro examinations demonstrated that different plant and fundamental oil removes repress bacterial species found in cutaneous diseases [159]. In vitro, chamomile essential oil and -bisabolol were found to have bactericidal and fungicidal activity (mostly against Gram-positive bacteria, *S. aureus*, *B. subtilis*, and the fungus *C. albicans*) [166].

Infections of the skin can also be treated with *A. cepa* and *R. officinalis*. *Malassezia furfur* (25 strains), *C. albicans* (18 strains), other *Candida* sp. (12 strains), and 35 strains of other dermatophyte species were investigated for antifungal activity using aqueous extracts from *A. cepa* (onion; AOE) and *A. sativum* (garlic; AGE). The findings revealed that onions and garlic could help treat fungal infections caused by pathogenic genera like *Candida, Malassezia*, and dermatophytes [167].

#### 7.1.4. Digestive Infection

*Vaccinium myrtillus* is, without a doubt, the most commonly suggested plant species for stomach illnesses (bilberry). A clinical preliminary demonstrates the adequacy of Jiechang combination, a conventional Chinese medicine herb, in the treatment of juvenile mycosis enteritis [159].

### 7.2. Phytotherapy in Viral Infection 

The flexible immunomodulator *Echinacea angustifolia* (tight leaf echinacea) is doubtlessly the best choice for treating the ordinary cold and hindering influenza disarrays. Other recurring viral diseases include infectious herpes simplex, and the appearance of acyclovir has made treatment more troublesome. Birch bark has been demonstrated to hinder acyclovir-sensitive and acyclovir-resistant bacteria [3,168].

### 7.3. Phytotherapy in Parasitosis 

Numerous parasite contaminations are dangerous to individuals’ wellbeing, but helminth infections are the most prevalent [169]. Ramson (*Allium ursinum* TM) accelerates digestion and aids in the elimination of intestinal worms.

Suppositories, on the other hand, may be suggested in pediatrics. *Nigella sativa* L. seeds (black cumin, fennel flower, and negrilla) have been utilized to treat a variety of clinical conditions, including helminth infections, epilepsy [170], and oral malodor prevention [171,172].

In helminth infections, promising disclosures have been obtained; another formula of significantly antiparasitic substance silver doped titanium dioxide nanoparticles (TiAgNps) and *Nigella sativa* L. basic oil is particularly incredible against Cutaneous Leishmaniasis [173].

## 8. Active Compounds from Medicinal Plants 

**Figure 2:**
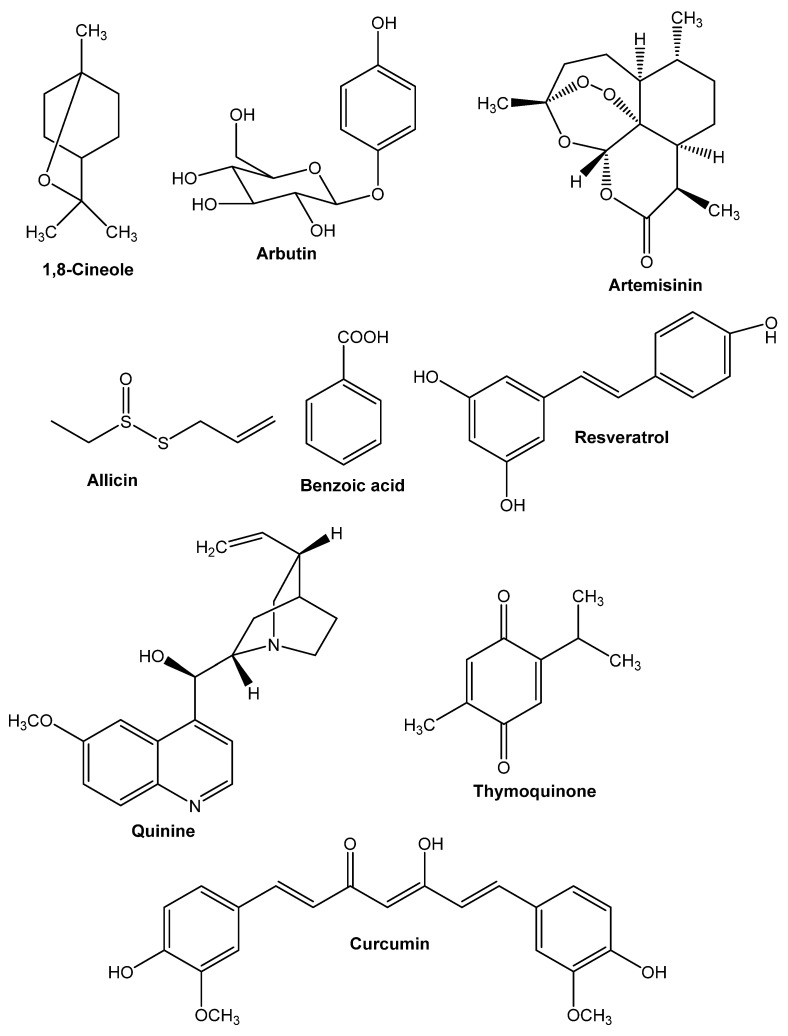
Secondary metabolites presented significant antimicrobial properties from medicinal plants and phytochemicals.

Some other secondary metabolites from medicinal plants show some major antimicrobial properties that are significant active compounds (Figure 2).

### 8.1. 1,8-Cineole

Numerous fundamental oils incorporate (ethyl-dimethyl-(3-sulfopropyl)azanium) (C_7_H_18_NO_3_S^+^) (PubChem CID 448830, for example, *E. globulus* (eucalyptus) oil, *Melaleuca alternifolia* (tea tree) oil, or *R. officinalis* oil (rosemary). It is one of the fundamental unique combinations in key oils, which clarifies their antibacterial properties [2]. It functions as an accelerator of penetration for topical application to the skin [16]. By temporarily disrupting the intercellular lipids in the skin’s stratum corneum, 1,8-cineole enables the penetration of otherwise less penetrable substances into the skin [174].

### 8.2. Arbutin

Plants from the Ericaceae, Asteraceae, and Rosaceae families contain arbutin ((2R,3S,4S,5R,6S)-2-(hydroxymethyl)-6-(4-hydroxyphenoxy)oxane-3,4,5-triol) (C_12_H_16_O_7_) (PubChem CID 440936). It explains the antiseptic qualities, although its interactions with bacteria, particularly the carcinogenic impact of its metabolite’s hydroquinone, are not well understood [175,176].

### 8.3. Allicin

(3-prop-2-enylsulfinylsulfanylprop-1-ene) (C_6_H_10_OS_2_) (PubChem CID 65036) belongs to the Alliaceae family and contains antibacterial and immunomodulatory properties, as well as numerous other health benefits [175].

The antibiotic activity of allicin was initially being thought to be mediated mainly through the inhibition of specific thiol-containing enzymes in bacteria via the fast interaction of thiosulfinates with thiol groups [3]. Allicin’s capacity to react with a model thiol molecule (L-cysteine) to create the S-thiolation product S-allyl mercapto cysteine was recently validated in a study [176].

### 8.4. Artemisinin

Artemisinin (C_15_H_22_O_5_) (PubChem CID 68826) is a semi-synthetic plant-derived chemical with a prolonged usage history in infectious illnesses. The dynamic part of *Artemisia annua* (sweet wormwood) is artemisinin, which has given the medical community new expectation [177,178]. The development of artemisinin-resistant *Plasmodium falciparum* explains why novel antimalarial is so essential. *Lupane triterpenes*, derived from *Buxus sempervirens* (common boxwood), have just been identified as the next generation of antimalarials, according to a recent study [179,180].

### 8.5. Benzoicacid

The antibacterial action of benzoic acid (C_7_H_6_O_2_) (PubChem CID 243), which is present in the fruits of *Vaccinium vitis-idaea* (lingonberry, cowberry, or bearberry), explains jam preservation [2].

### 8.6. Curcumin

Curcumin ((1E,6E)-1,7-bis(4-hydroxy-3-methoxyphenyl)hepta-1,6-diene-3,5-dione) (C_21_H_20_O_6_) (PubChem CID 969516), a functioning blend extricated from fragrant *Curcuma aromatica* (wild turmeric), not only an amazing malignant growth avoidance agent, calming, and anticancer fixing, yet it furthermore has antiparasitic action in vitro [2]. Curcumin is effective at suppressing both acute and chronic inflammation. It inhibits inflammation by decreasing histamine levels and possibly increasing adrenal glands’ natural cortisone production [8]. Additionally, curcumin demonstrated anti-inflammatory activity in vitro against human vascular cells. Curcumin exerts anti-inflammatory activity by inhibiting the inflammatory response of TNF-α stimulated human endothelial cells by interfering with NF-κB. Additionally, curcumin inhibits platelet-derived growth factor (PDGF) [181].

### 8.7. Quinine

(R)-[(2S,4S,5R)-5-ethenyl-1-azabicyclo[2.2.2]octan-2-yl]quinine(R)-[(2S,4S,5R)-5-ethenyl-1-azabicyclo[2.2.2]octan-2-yl]quinine(R)-[(2 Cinchona produces-(6-methoxyquinolin-4-yl)methanol) (C_20_H_24_O_2_N_2_ or C_20_H_24_N_2_O_2_) (PubChem CID 3034034). This compound was considered the primary treatment for malaria for quite a long time, although alternative plant-based active chemicals have now proven their worth [2]. Additionally, numerous investigations have indicated that quinine exhibits antibacterial effects. Quinine was discovered to be bactericidal when *E. coli*, *K. pneumonia,* and *S. aureus* were inhibited [182].

### 8.8. Resveratrol

Resveratrol(5-[(E)-2-(4-hydroxyphenyl)ethenyl]benzene-1,3-diol) (C_14_H_12_O_3_)(PubChem CID 445154) is an antioxidant that also possesses antibacterial effects [183]. Resveratrol inhibits the ATP synthase enzyme in a variety of bacteria [17].

### 8.9. Thymoquinone

Thymoquinone (2-methyl-5-propane-2-ylcyclohexa-2,5-diene-1,4-dione) (C_10_H_12_O_2_) (PubChem CID 1028) is an essential oil, and is also the major active component in *N. sativa* L. seeds. It has anti-inflammatory properties as well as being a flexible immunomodulator with unique pathways recently revealed. The valuable results of *N. sativa* L. seeds essential oil in viral diseases can be clarified by its contribution in initiating cell invulnerability by rousing CD4+ T cells and producing -interferon [184,185].

## 9. Chemical Compounds Having Antimicrobial Properties Derived from Marine Source

### 9.1. Terpenoids

**Figure 3:**
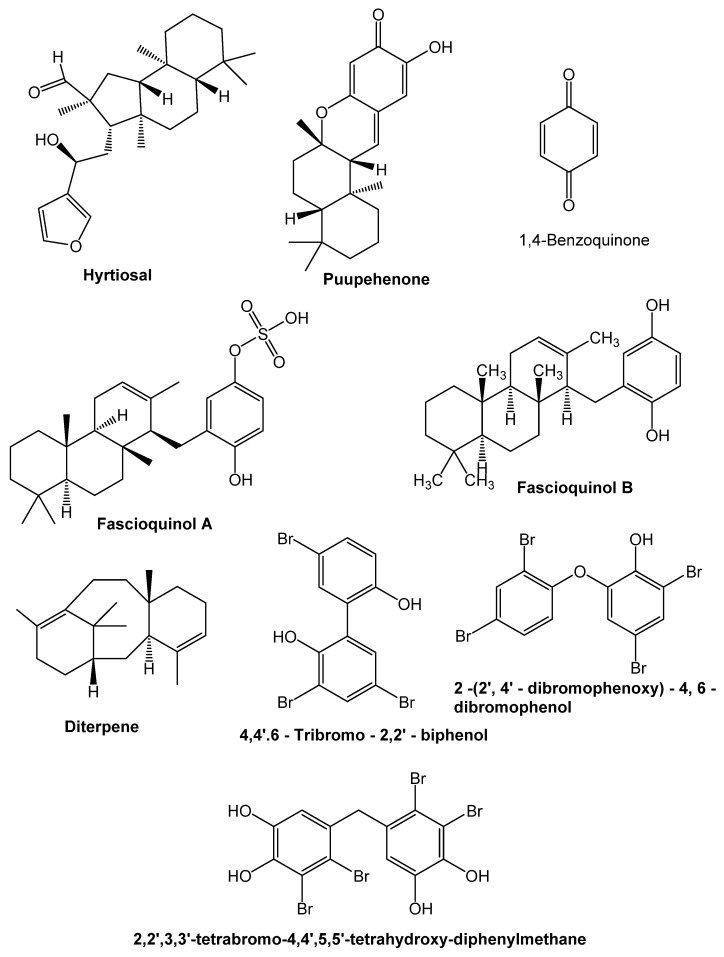
Secondary metabolites presented significant antimicrobial properties from terpenoids and phenolic compounds.

Terpenoids are bountiful in higher plants and are widely spread in nature. Uncommon terpenoids can likewise be found in bounty in marine species. Terpenoids are considered phytoalexins, bug antifeedants and anti-agents, fertilization attractants, herbivore protection specialists, pheromones, allelochemicals, plant chemicals, and sign atoms, and they have ruled the subject of synthetic biology. As per current artificial information, the elementary classes of antibacterial and antiviral terpenoids found in the sea climate incorporate sesterterpenoids, sesquiterpenoids, and meroterpenoids. Marine sesterterpenoids are plentiful, particularly in wipes, and they have a wide scope of biological activities, for instance, antibacterial and antiviral impacts [186]. Lee et al. [187] secluded seven sesterterpene sulfates from the tropical wipe *Dysidea* sp. likewise contemplated their prohibiting activity against *C. albicans* isocitrate lyase. A large portion of blends was discovered to be incredible isocitrate lyase inhibitors with the antibacterial movement against *B. subtilis* and *P. vulgaris.* Hyrtiosal (Figure 3) got from the marine wipe *Hyrtios erectus*, is another bioactive sesterterpenoid that represses HIV integrase (IN) confining to viral DNA through a novel inhibitor restricting area [188]. The way that hyrtiosal could tie HIV N-terminal space at Ser17, Trp19, and Lys34 created such hyrtiosal-instigated viral DNA/IN hindrance, as indicated by a unique sub-atomic investigation matched with a site-coordinated mutagenesis strategy. Since hyrtiosal was as recently demonstrated to be a protein tyrosine phosphatase 1B inhibitor, this exploration could give data on numerous objectives to this marine regular substance. Many fascinating dynamic sesquiterpene–quinones/–hydroquinones are being found in marine wipes. The 1,4-benzoquinone moiety is found in a wide scope of accumulates that have gotten a great deal of interest because of their wide scope of natural exercises, including antibacterial and antiviral attributes. Puupehanol, alongside the known synthetic substances puupehenone and chloropuupehenone, is a novel sesquiterpene–dihydroquinone subordinate discovered from ocean wipe *Hyrtios* sp. that is liable for the antifungal action found in the wipe separate [189]. Puupehenone has the most grounded suppressing development against *Cryptococcus neoformans* and *Candida krusei* of the overall large number of substances reviewed, with MICs going from 1.25 to 2.50 g/mL, independently. Other antimicrobial sesquiterpenoid-quinones found in marine wipes include nakijiquinones G-I, isolated from Okinawan marine wipes of the Spongilidae family, and new sesquiterpenoid–hydroquinones from the marine wipe Dysidea Arenaria, which had a moderate inhibitory effect on HIV pivot transcriptase (RT) [190]. 

Other antimicrobial terpenoids isolated from ocean wipes included meroterpenoids, according to reports. Zhang et al. extracted fascioquinols A–F as naturally dynamic meroterpenes from significant water southern Australian ocean wipes during a study to discover new antimicrobial experts from marine living things. Fasciospongia sp. is a species of Fasciospongia. Fascioquinols B, C, and D result from fascioquinol A’s destructive interceded hydrolysis/cyclization delayed consequences. Two of these combinations, facioloquinol A and B, showed promising Gram (+) unequivocal antibacterial activity against *S. aureus* (IC_50_ 0.9–2.5 M) and *B. subtilis* (IC_50_ 0.3–7 M). Novel Caledonia’s distant ocean wipe yielded four new meroterpenes, alisiaquinones A-C, and alisiaquinol. The blends inhibited the plasmodial kinase Pfnek-1 and a protein farnesyltransferase in the M reach, as well as different chloroquine-sensitive and –safe Plasmodium falciparum strains. Tropical ocean diterpenes and diterpene isonitriles wipe Another antimicrobial terpenoid found in marine wipes is *Cymbastela hooperi* [191].

### 9.2. Phenolic Compounds 

Phenols are the most widely perceived sort of auxiliary metabolite found in plants. They range from straightforward designs with one aromatic ring to exceptionally complex polymeric substances and can be found in numerous classes of typical blends with sweet-smelling moieties. In the marine climate, phenolic compounds with halogen can be found frequently. An enormous number of studies on the antimicrobial action of phenolic compounds disconnected from ocean wipes, principally antibacterial movement, have been led as of late (Figure 3). 2-(2′,4′-dibromophenoxy)-4,6-dibromophenol from an ocean wipe in vitro antibacterial movement was found in *Dysidea granulosa* gathered off the shore of the Lakshadweep Islands in the Indian Ocean, especially against methicillin-safe and *touchy S. aureus*; vancomycin-safe, and delicate *Enterococci*; and *Bacillus* sp. A tale polybrominated diphenyl ether, which was recognized from another Dysideaspecies from the Federated States of Micronesia [192]. In the hyphae arrangement hindrance measure, these medications showed inhibitory movement against *Streptomyces 85E.* The Indonesian wipe *Lamellodysidea herbacea* was additionally displayed to contain these synthetics [193]. Antimicrobial viability against *Bacillus subtilis* was exhibited by these metabolites. Incorporating two phenolic hydroxyl gatherings and bromines at C-2 and C-5 is vital for presenting antibacterial movement, as indicated by investigations of construction action communications. Other marine creatures and microorganisms, like red–green growth and microbes, have been found to contain bromophenol synthetics. In the wake of distinguishing movement in marine concentrates, a portion of these synthetics were secluded utilizing bioassay-directed fractionation. Gracious et al. [194] gathered *Odonthalia corymbifera* in the journey for naturally dynamic fixings in marine green growth and found that its unrefined concentrates have antibacterial activity against an assortment of organisms. The crude concentrate was isolated utilizing a bioassay-directed strategy, yielding various bromophenol compounds. The 2,2′,3,3′-tetrabromo-4,4′,5,5′-tetrahydroxy diphenylmethane subsidiary was the most dynamic against *Candida albicans*, *Aspergillus fumigatus*, *Trichophyton rubrum*, and *Trichophyton mentagrophytes* among the secluded normal items. 4,4′,6-tribromo-2,2′-biphenol, obtained from a concentrate of a marine *Pseudoalteromonas sp*. CMMED 290 showed noteworthy antibacterial development against *methicillin*-safe *Staphylococcus aureus*. Isnansetyo and Kamei recovered 2,2′,3-tribromo-biphenyl-4,4′-dicarboxylic destructive from another *Pseudoalteromonas* sp. the marine bacterium *Pseudoalteromonas phenolica* O-BC30T. This was found to threaten methicillin-safe Staphylococcus aureus action against all ten clinical isolates, with MIC regards going from 1 to 4 g/mL. The combination was convincing against *B. subtilis* and *Enterococcus serolicida*, yet not Gram (−) minuscule life forms or development. These disclosures demonstrated that this bromophenyl compound has strong in vitro movement against methicillin-safe *S. aureus* and could be used as a lead particle to improve novel antimicrobials. Other antibacterial bromophenyl compounds have been found from the INH strain of the marine bacterium *Pseudoalteromonas haloplanktis* [195].

### 9.3. Alkaloids 

**Figure 4:**
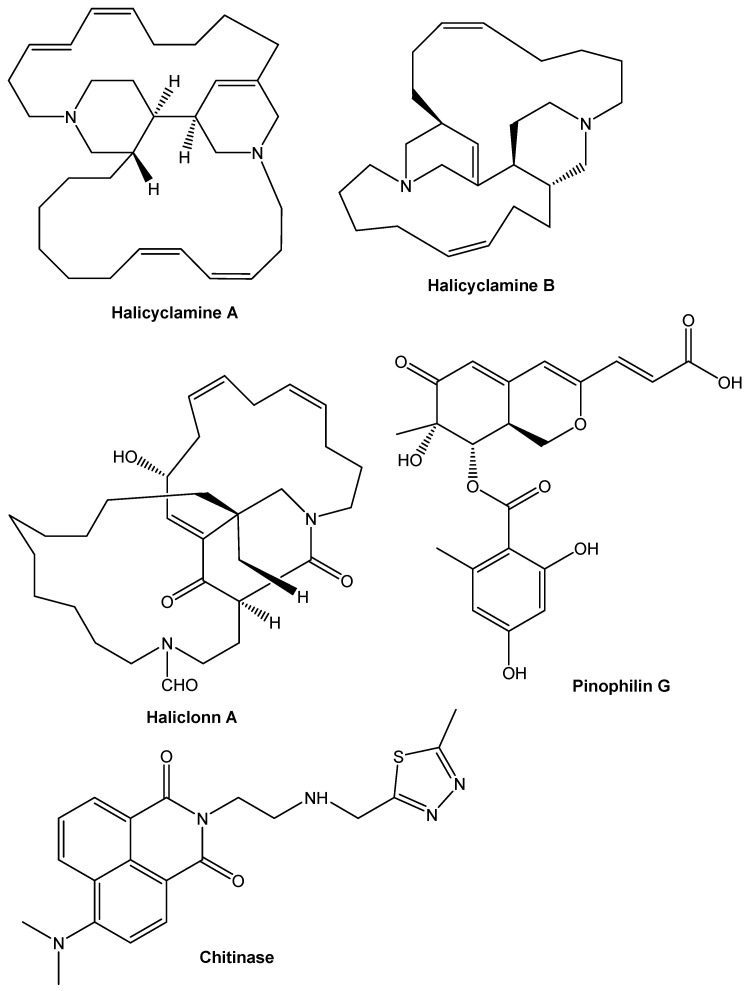
Secondary metabolites presented significant antimicrobial properties from alkaloids, polysaccharides, and fungi.

Alkaloids with particular synthetic highlights and conspicuous substance action have been found in marine organic entities and microorganisms, recommending that they could be helpful as lead structures to improve novel prescriptions (Figure 4). Antimicrobial and antiviral exercises are among the expected pharmacological impacts of a few of these substances. Many intriguing antimicrobial dynamic nitrogen-containing heterocyclic mixtures, such as alkylpiperidine, bromopyrrole, and pyrroloiminoquinone alkaloids, are found in marine wipes. *Halicyclamine A* was rediscovered as a lead for hostile to tuberculosis specialist from a marine wipe of *Haliclona* sp. on the direction of the created biological assay in the journey for antimicrobial medications against torpid *Mycobacterium tuberculosis* [196]. *Halicyclamine A* repressed the improvement of *Mycobacterium smegmatis, Mycobacterium Bovis,* and *Mycobacterium TB* with MICs going from 1 to 5 g/mL in both vigorous and hypoxic conditions, causing lethargy. Halicyclamine A’s development inhibitory movement was bactericidal, and it did not cross-oppose isoniazid, ethambutol, rifampicin, or streptomycin, which are right now utilized as anti-tubercular drugs. This wipe, as of late, gave 22-hydroxyhaliclonacyclamine B, a novel tetracyclic alkyl piperidine alkaloid, just as two familiar alkaloids, haliclonacyclamine An and B, as against lethargic mycobacterial compounds [197]. Since 22-hydroxyhaliclonacyclamine B has decreased antibacterial activity against *M. tuberculosis*, it may be extrapolated that the 22-hydroxy gathering in position one was found to decrease hostile to mycobacterial action in examinations of construction action associations. Haliclonin A, another antimicrobial alkaloid from *Haliclona* sp., was found to have antibacterial activity against an assortment of microbial strains [198]. 

Bromopyrrole alkaloids, which are familiar among the most continuous metabolites found in these species, are additionally antibacterial alkaloids found in marine wipes. These antimicrobial segments, for example, nagelamides Q, R, J, K, L, M, and N, have been recognized from the wipe *Agelas* sp. [199,200,201]. *Monanchora unguifera* has been displayed to contain various polycyclic guanidine alkaloids with antiviral and antibacterial properties [202]. Batzelladine alkaloids such as 16-hydroxycrambescidin 359; batzelladines K, L, M, and N; ptilomycalin A; crambescidine 800; batzelladine C; and dehydrobatzelladine C were recovered from this Caribbean plant. The mixtures were found to have antiretroviral action against HIV and AIDS’s shrewd diseases microbes. Merobatzelladines An and B have, as of late, been distinguished as antibacterial constituents from this marine wipe [203,204].

### 9.4. Polysaccharides

The immense number of polysaccharides are taken from marine plants and creatures, or made by ocean microbes and parasites, which implies that the sector of marine polysaccharides is continually expanding [205]. The antimicrobial and antiviral movement was seen in a portion of these marine polysaccharides (Figure 4). The antiviral part (hostile to HSV-1) of the acidic polysaccharide nostoflan was separated from the scrumptious blue-green algae *Nostoc flagelliforme* [206]. In time-of-addition studies, early events such as virus binding and/or penetration were revealed to be the most sensitive stage of viral replication to nostoflan. Separate virus binding and penetration assays were performed to see how much nostoflan is involved in these processes.

The findings suggest that the antiherpetic activity of nostoflan is due to the suppression of virus binding to, but not penetration into, host cells. Infection restricting and infiltration tests were done independently to determine the degree to which nostoflan might be engaged with these cycles. Another antiviral polysaccharide of marine origin is a lectin obtained from the filamentous *cyanobacterium Oscillatoria agardhii* NIES-204, which smothers HIV propagation in MT-4 cells [207]. The antifungal movement was found in marine polysaccharides, for example, a chitinase segregated from an ocean *Streptomyces* sp. DA11 connected with the South China ocean wipe *Craniella australiensis* [173], which expressed antifungal action against *Aspergillus niger* and *C. albicans.* The wipe’s microbial symbiont might support chitin breakdown and antifungal safeguard with chitinase action [173].

### 9.5. Fatty Acids 

Unsaturated fats containing at least two methylene-intruded on twofold securities are significant for excellent cell work. Their organic pertinence in explicit clinical issues normal in Western culture, like heftiness and cardiovascular sickness, has prompted their utilization in biomedical and nutraceutical research. Marine unsaturated fats are intriguing due to the different jobs and natural highlights they play in marine animals’ cells. A portion of these unsaturated fats has been displayed to have significant natural highlights, like antibacterial and antiviral action. The calcareous wipe *Paragrantia* cf. *waguensis* has yielded a novel acetylenic unsaturated fat [208]. With MICs of 64 and 128 g/mL, the substance exhibited antibacterial advancement against *S. aureus* and *E. coli*, autonomously. Brominated unsaturated fats from an ocean wipe gathered in Papua New Guinea [209] and motualevic acids A–F separated from the wipe *Siliquaria spongia* sp., which stifles the improvement of *S. aureus* and its methicillin-safe strains are instances of other antimicrobial unsaturated fats from seawater wipes [210]. Marine algae have also been used to isolate antimicrobial fatty acids. Extracts from the marine diatom *Phaeodactylum tricornutum* have been reported to have antibacterial properties. Desbois et al. [211] antibacterial compounds like the monounsaturated fatty acid (9Z)—hexadecenoic acid and the relatively uncommon polyunsaturated fatty acid (6Z,9Z,12Z)—hexadecatrienoic acid were discovered and recognized as being responsible for this activity. Both compounds are effective against Gram (+) bacteria, with *Listonella anguillarum*, a Gram (−) marine pathogen, showing an extra inhibitory impact. The first chemical kills bacteria quickly at μM concentrations and is extremely effective against multidrug-resistant *S. aureus*. Eicosapentaenoic acid, a novel antibacterial fatty acid produced by this diatom, is efficacious against a wide spectrum of Gram (+) and Gram (−) bacteria, including multi resistant *S. aureus* [212].

The culture extract of *A. niger* EN-13, an endophytic fungus isolated from the marine brown algae *Colpomenia sinuosa* [213], yielded Asperamides A and B, a sphingolipid and its related glycosphingolipid with a previously unreported 9-methyl-C20-sphingosine moiety. Asperamide A showed antifungal activity against *C. albicans* in the antifungal assay.

### 9.6. Fungi 

Comazaphilones C–E (30–32), azaphilone subsidiaries, were distinguished from Penicillium cooperative QSD-17, which was recuperated from oceanic residue in the southern China Sea. Antibacterial action was seen in intensifies 30–32, with MIC esteems going from 16 to 64 lg/mL [214]. A marine-determined parasite strain, *Penicillium* sp., was utilized to create 7-O-acetylsecopenicillide C (33) MA-37 was segregated from the marine mangrove plant *Bruguiera gymnorrhiza*’s rhizospheric soil test. Compound 33 hindered the development of *Micrococcus luteus* and *E. coli*, individually, having MIC upsides of 64 and 16 L g/mL [215,216]. *Penicillium* sp. ML226 was found in a silt test taken in the Fu Gong mangrove zone of Long Hai, Taiwan Strait, China, and created penicitrinols J (34) and K. (35). The two mixtures (34 and 35) were antibacterial against *S. aureus* CMCC26003, with inhibitory zones of 4 and 3 mm, separately, at 20 mg/plate [216]. *P. spinophilin* SD-272 was detached from a dregs test in the Pearl River, South China Sea, and it is anything but A (36), which restrained *E. coli* with a 10 mm restraint zone at 20 L g/circle [217]. Herqueidiketal (37), another synthetic with an exceptionally oxidized naphthoquinone fraction confined from the growth *Penicillium* sp. F011, showed antibacterial movement against *S. aureus* with an IC_50_ of 23.6 IM. *Pinophilin G* (38), an azaphilone subordinate separated from the marine-determined growth *P. spinophilin*, was found to have antibacterial action against *Vibrio anguillarum* with an MIC worth of 25.0 mol/L (Figure 4) (172). *Penicillium* sp. SCSIO of 101 was separated from an oceanic dregs test acquired from the South China Sea and was displayed for penicacid D (39), an antibacterial specialist with an MIC worth of 641 g/mL against *E. coli* [218].

### 9.7. Actinobacteria

**Figure 5:**
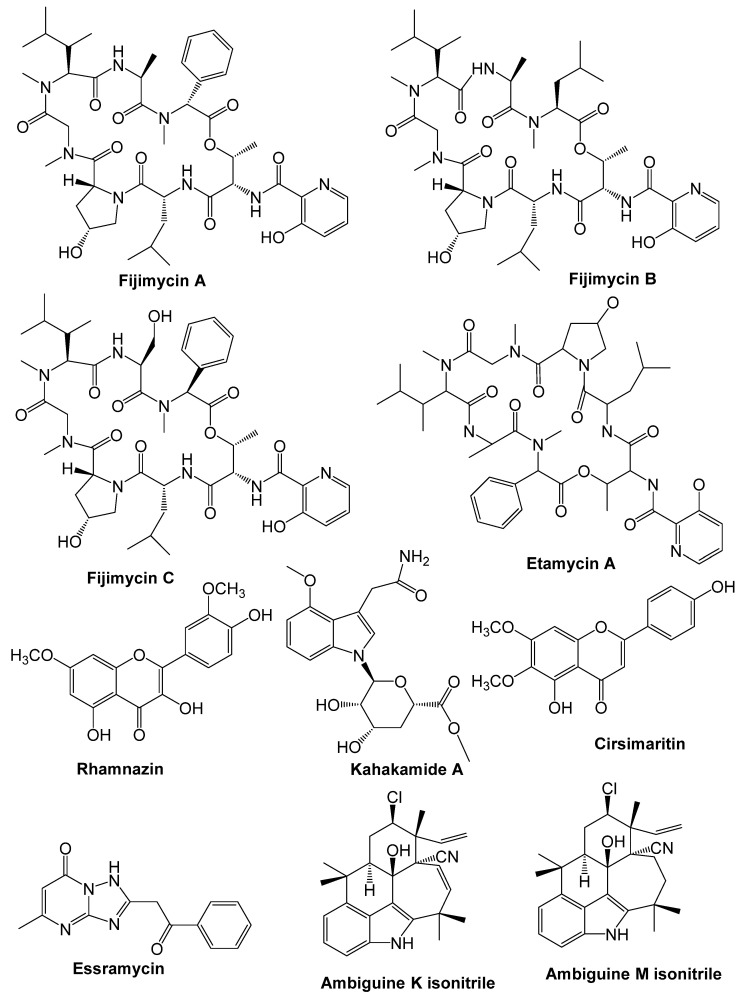
Secondary metabolites presented significant antimicrobial properties from actinobacteria and Cyanobacteria.

Actinobacteria, a phylum of germs, is found in an assortment of traditional settings. The sort *Streptomyces* creates maximum microbial-determined biologically active mixtures combined by agents of the request Actinomycetes, which are Gram-positive, non-motile, oxygen- consuming microorganisms with high G C substance in their DNA (70–80 percent) and high phenotypic variety (Figure 5). Low natural matter focus, high hydrostatic pressing factor, high NaCl fixation, and low temperature are, for the most part, present in a profound marine living space. Thus, it is conceivable that marine Actinomycetes have interesting qualities that have not been seen in earthly Actinomycetes [219]. A few marine actinobacteria have effectively been displayed to make unmistakable auxiliary metabolites [220]. Actinomycetes have been broadly examined as antibacterial substance producers such as synthetic antimicrobial compounds found in marine strains. *Streptomyces* sp. was segregated from ocean residue and displayed to create the anti-toxin synthetics fijimycins A–C (intensifies 1–3) and etamycin A (4), which had considerable antibacterial action against MRSA strains (86). In the circle dispersion examine, the unprecedented neosidomycin metabolite kahakamide A (5) has antibacterial activity against *Bacillus subtilis* [221]. Heronamycin is delivered by other *Streptomyces* species gathered from ocean garbage (6, a benzothazine ansamycin). This synthetic displayed having unobtrusive antibacterial action against two strains of *B. subtilis* [222]. Lynamicins A–E (7–11), chlorinated bisindole pyrroles were distinguished from sea silt-related actinomycetes. Medication safe microbes, including MRSA and vancomycin-safe *Enterococcus faecium* (VRE) are helpless to these mixtures [223]. Novel antimicrobial synthetic substances, including three benzopyrone subordinates, 7-methyl coumarin, and two flavonoids, rhamnazin and cirsimaritin, were distinguished from a *Streptomyces* sp. disconnected from a sea silt [224]. Essramycin (12), for instance, has been exhibited to have antibacterial viability against *P. aeruginosa*, *E. coli*, and *Micrococcus luteus* [224,225].

### 9.8. Cyanobacteria

Cyanophyta is a solitary bacterial phylum that gets energy through photosynthesis and is the lone photosynthetic prokaryotes equipped for creating oxygen and engrossing CO_2_ [226]. Although some Cyanobacteria are hard to develop, this gathering of microorganisms accumulates consideration in essential exploration because of its shortage. Unicellular and filamentous cyanobacteria have been assembled throughout the long term, portrayed morphologically and microscopically, and developed in axenic societies utilizing lumbering techniques. The present metagenomics structure may help accomplish unadulterated culture necessities [227] for additional therapeutic property research **(Figure 5**). Cyanobacteria are an incredible non-customary wellspring of mixtures with a splendid and cheerful future in prescription advancement for various illnesses [228]. Indeed, compounds confined from these microorganisms have been displayed to have antibacterial, antifungal, antiviral, anticancer, antiplasmodium, and antialgae activity. It additionally seems to have immunosuppressive properties [229]. Ambiguine-K, Ambiguine K-O isonitriles, and M isonitriles have been segregated from the oceanic cyanobacterium *Fischerella ambigua* (UTEX 1903) and show critical antibacterial action against *Mycobacterium TB* [230]. Lyngbyoic corrosive is a cyclopropane-containing unsaturated fat that has been separated from the marine *cyanobacterium Lyngbya* and can disturb *P. aeruginosa* majority detecting. This particle envelops over 260 marine synthetic compounds and is right now going through preclinical testing, including certain macrolactins created by *Bacillus* spp., napyradiomycins, and dixiamycins delivered by *Streptomyces* spp. [71,225].

## 10. Conclusions and Future Prospects

Antimicrobials have become important in contemporary life; the wonders of these drugs display the usefulness of the natural world. However, the inappropriate use and over-prescription of medications leads to multi-drug resistance (MDR) and cross-resistance to other drugs, which have become worldwide concerns. This emerging problem has ushered in the Era of Responsibility. Warning bells can be heard from every quarter. There is an urgent need to discover some novel alternatives to help combat this significant problem. Many studies are currently in process about the antimicrobial properties of natural products. The current literature in this area indicates an urgent need for a coordinated effort for meaningful research and the discovery of novel alternatives by exploring natural products.

This investigation shares a plethora of information regarding the phytocomplexes and the mode of action of pure antimicrobials isolated and purified from the potential medicinal plants. These constituents also unlock the opportunities to design new nutraceuticals or other effective drugs. However, future investigation should explore the additional bioactivity of the corresponding genus and species to discover new therapeutics.

## Data Availability

Not applicable.
